# Hydroxylated transformation products obtained after UV irradiation of the current-use brominated flame retardants hexabromobenzene, pentabromotoluene, and pentabromoethylbenzene

**DOI:** 10.1007/s11356-023-30566-w

**Published:** 2023-11-02

**Authors:** Alexandra Klimm, Walter Vetter

**Affiliations:** University of Hohenheim, Institute of Food Chemistry (170b), Garbenstraße 28, D-70599 Stuttgart, Germany

**Keywords:** Brominated flame retardant, Photolysis, Metabolite, Hydroxylated transformation product, Bromophenol, Brominated dihydroxybenzene, Brominated alkylphenol

## Abstract

Hexabromobenzene (HBB), pentabromotoluene (PBT), and pentabromoethylbenzene (PBEB) are current-use brominated flame retardants (cuBFRs) which have been repeatedly detected in environmental samples. Since information on hydroxylated transformation products (OH-TPs) was scarcely available, the three polybrominated compounds were UV irradiated for 10 min in benzotrifluoride. Fractionation on silica gel enabled the separate collection and identification of OH-TPs. For more insights, aliquots of the separated OH-TPs were UV irradiated for another 50 min (60 min total UV irradiation time). The present investigation of polar UV irradiation products of HBB, PBT, and PBEB was successful in each case. Altogether, eight bromophenols were detected in the case of HBB (three Br_3_-, four Br_4_-, and one Br_5_-isomer), and nine OH-TPs were observed in the case of PBT/PBEB (six Br_3_- and three Br_4_-congeners). In either case, Br➔OH exchange was more relevant than H➔OH exchange. Also, such exchange was most relevant in *meta*- and *ortho*-positions. As a further point, and in agreement with other studies, the transformation rate decreased with decreasing degree of bromination. UV irradiation of HBB additionally resulted in the formation of tri- and tetrabrominated dihydroxylated compounds (brominated diphenols) that were subsequently identified. These dihydroxylated transformation products were found to be more stable than OH-TPs.

**Supplementary Information:**

The online version contains supplementary material available at 10.1007/s11356-023-30566-w.

## Introduction

Brominated flame retardants (BFRs) are one of different product classes that were and are currently applied to prevent fires and to warrant safety in homes and vehicles (Birnbaum and Staskal 2004; Papachlimitzou et al. 2012; Besis et al. 2017; Włuka et al. 2020). However, detrimental environmental properties including persistency and toxic effects of several first generation BFRs like polybrominated diphenyl ethers (PBDEs), polybrominated biphenyls (PBBs), and hexabromocyclododecane (HBCD) have resulted in their ban in the signing countries of the Stockholm Convention on persistent organic pollutants (POPs) (Da Chen et al. 2015; Yu et al. 2016; D'Silva et al. 2004; Chang et al. 2021). Consequently, these BFRs had to be replaced by so-called current-use flame retardants (cuBFRs) (Egebäck et al. 2012; Wemken et al. 2019; Wang et al. 2012). This pool of flame curtailing additives includes a few comparably small monoaromatic molecules such as hexabromobenzene (HBB), pentabromotoluene (PBT), and pentabromoethylbenzene (PBEB) that are applied in various industrial products like electronic and plastic goods, paper, wood, and textiles (Covaci et al. 2011; Kim et al. 2014; Morin et al. 2017; Ling et al. 2019; Chen et al. 2019).

Similar to PBDEs, PBBs, and HBCD, these monoaromatic cuBFRs have also been detected in various environmental samples like air and dust (Covaci et al. 2011; Möller et al. 2011; Zhao et al. 2021), sediments (Gauthier et al. 2007; Wang et al. 2017), mammals (Montie et al. 2010; Berger et al. 2023), bird’s eggs (Gauthier et al. 2009; Vetter et al. 2017), homes (Gallistl et al. 2017; Bendig et al. 2013), and human hair (Zhao et al. 2021). In addition, several UV transformation products, formed by hydrodebromination (a.k.a. reductive debromination), were detected in laboratory experiments (Klimm and Vetter 2021; Klimm et al. 2019). Compared to that, almost no knowledge about photolytically formed hydroxylated transformation products (OH-TPs) can be found in the literature (Klimm and Vetter 2021).

The goal of the present study was to conduct UV irradiation experiments with HBB, PBT, and PBEB in the laboratory. Potentially present OH-TPs were then separated from their less polar precursors and hydrodebromination products. Evaluation of the product spectrum was supplemented by a second UV irradiation performed with the separated OH-TP fraction. In this way, the precursors were removed and the fate of the OH-TPs could be monitored and intercompared. Evaluation of the product spectrum by gas chromatography with electron capture negative ion mass spectrometry (GC/ECNI-MS) could be based on structure information available from a recent synthesis study of OH-TPs (Klimm and Vetter 2022).

## Materials and methods

### Chemicals and standards

Pure (>98%) 1,2,3,4,5-pentabromotoluene (PBT), 1,2,3,4,5,6-hexabromobenzene (HBB), and 1,2,3,4,5-pentabromoethylbenzene (PBEB) were obtained from Santa Cruz Biotechnology (Dallas, TC, USA). The internal standard 4,6-dibromo-2-(2′,4′-dibromo)-phenoxyanisole (2′-MeO-BDE 68, BC-2) was previously synthesized in our working group (Vetter et al. 2003). Pyridine (≥99.9%, distilled before use), acetic anhydride (≥99%), ethyl acetate (anhydrous, 99.8%), *α,α,α*-trifluorotoluene (≥99%, anhydrous; benzotrifluoride, BTF), and silica gel 60 were obtained from Sigma-Aldrich (Steinheim, Germany). Toluene (≥99.8%) came from LGC Standards (Wesel, Germany) and *n*-hexane (≥95%, for pesticide residue analysis) was purchased from Th. Geyer (Renningen, Germany). Fluka Analytics (Seelze, Germany) delivered 2,2,4-trimethylpentane (*iso*-octane, 99.5%) while sodium sulfate (≥99%, anhydrous) was acquired from Carl Roth (Karlsruhe, Germany).

### cuBFR standard solutions

HBB (4.04 mg), PBT (4.01 mg), and PBEB (4.25 mg) (chemical structures illustrated in Fig. S1) were dissolved in 5 mL BTF, respectively, and if necessary, sonicated for 15 min (concentrations ~800 ng/μL). The concentrations were about 3–4 times higher than those used by Mas et al. (2011) in UV irradiation experiments with bromophenols. The slightly higher amounts were considered appropriate given the fact that the share of OH-TPs was excepted to be low in comparison with the precursors. Parallel experiments were carried out with toluene (4.09 mg HBB, 3.96 mg PBT, and 4.50 mg PBEB in 5 mL toluene). Contrary to the reaction rate of hydrodebromination (toluene>> BTF (Klimm and Vetter 2021)), the variety of OH-TPs was much higher in BTF although the main products were the same in both solvents (data not shown). Since also the amounts of OH-TPs were ~3–40 times lower in toluene, only results obtained in BTF will be reported in the following.

### Initial UV irradiation of cuBFRs (1st UV irradiation, 10 min)

In a first step, cuBFR sample solutions were UV irradiated for 10 min, because this condition provided the highest amounts of OH-TPs with lowest formation of other by-products according to initial tests (data not shown). UV irradiation experiments were conducted in a 1.2-mL quartz cell using a 150 W medium-pressure mercury vapor lamp (TG 150, Heraeus Noblelight, Hanau, Germany) as the light source as presented before (Klimm and Vetter 2021). Due to the limited capacity of the quartz cell, five 1-mL aliquots of the entire sample solutions (5 mL, see previous section) were UV irradiated for 10 min, pooled and fractionated by column chromatography (see next section). Accordingly, the resulting sample pool presented the mean value of a fivefold determination.

### Fractionation of different product classes of UV transformation products by column chromatography on activated silica gel

OH-TPs in the solutions of UV irradiated pools of HBB, PBT, and PBEB (see previous step) were separated from the remaining share of the cuBFRs and their hydrodebromination products of the same polarity, as well as from potential solvent adducts (Klimm and Vetter 2021). The fractionation was performed in glass columns (30 cm length, 1.0 cm i.d.) equipped with a plug of glass wool, 8 g activated silica gel, and finally 1 g anhydrous Na_2_SO_4_ on top (Vetter et al. 2017). After conditioning with ~60 mL *n*-hexane, the UV irradiated sample solutions (previously condensed to ~1.0 mL) were loaded onto the column. The remaining share of cuBFRs and their hydrodebrominated transformation products were eluted with 48 mL *n*-hexane into silica gel fraction F1. Brominated adducts resulting from reaction with the solvent were eluted with 50 mL *n*-hexane/ethyl acetate (9:1 *v/v*) into silica gel fraction F2. Finally, the target compounds, OH-TPs with different degrees of bromination, were gained with 50 mL ethyl acetate (silica gel fraction F3). The recovery rate of silica gel fraction F3 (~97%) was exemplarily tested with pentabromophenol. Silica gel fractions F1–F3 were individually collected in 100-mL round bottom flasks. Corresponding results of fractions F1 and F2 have already been described before (Klimm and Vetter 2021). Silica gel fraction F3 was evaporated to ~2 mL. The solvent was removed at 40 °C by a gentle stream of nitrogen and re-dissolved in 2.5 mL BTF.

### Subsequent UV irradiation of aliquots of silica gel fraction F3 of the cuBFRs (2nd UV irradiation)

Silica gel fraction F3 (target compounds: OH-TPs, see previous step) of the respective cuBFR sample from the 1st UV irradiation (10 min, see above) was UV irradiated for a second time for 50 min (total UV irradiation time: 60 min). For this purpose, a 400-μL aliquot of the final solution of fraction F3 (16% of the samples) was removed and evaporated to dryness by a gentle stream of nitrogen, and the residue was taken up in 2.5 mL BTF. After 10, 15, 20, 30, 45, and 60 min of UV irradiation (same light source and conditions as shown above), respectively, aliquots of 50 μL were removed and placed in 1.5-mL amber vials with 200-μL micro-inserts. Since the sample solutions of silica gel fraction F3 had already been UV irradiated for 10 min during the 1st treatment, the starting point of the 2nd UV irradiation was assigned to 10 min UV irradiation time instead of 0 min. After adding the internal standard BC-2 (5.05 ng in 5 μL), samples were measured by GC/ECNI-MS. All 2nd irradiation experiments were performed in duplicate.

### Verification by acetylation of active oxygen sites (OH groups) in the compounds in silica gel fraction F3 of the UV irradiated cuBFRs

Aliquots (50 μL) of re-dissolved sample solution of silica gel fraction F3 (2% of the samples, see above) were transferred in 1.5-mL amber vials, and the solvent was removed at 40 °C by means of a gentle stream of nitrogen. Distilled pyridine (100 μL) and 100 μL acetic anhydride were added and the closed vials were heated to 60 °C for 1 h (Krauß et al. 2020). The solvent was again removed (see above) and the residue was re-dissolved in 50 μL BTF. These solutions were directly subjected to GC/ECNI-MS analysis.

### GC/ECNI-MS analysis

Operating conditions were set as described before on a 7890/5975C MSD system (Agilent, Waldbronn, Germany) (Klimm et al. 2019). In brief, the instrument was equipped with a 30 m × 0.25 mm i.d., 0.25 μm d_f_ Optima 5 MS capillary column (Macherey-Nagel, Düren, Germany). Data was recorded both in full scan (*m/z* 50–800) or selected ion monitoring (SIM) mode (Tab. S1, Supplementary Information). If not stated differently, sample solutions were evaluated in SIM mode.

### Short-term nomenclature of transformation products (TPs)

According to IUPAC rules, the numbering of OH-TPs was based on the phenolic backbones, hence the OH group was assigned to C1 (priority 1) (Klimm and Vetter 2022). However, in the case of PBT and PBEB, the alkyl branch (Me for brominated toluenes and Et for brominated ethylbenzenes) was set to priority 2, followed by listing the corresponding bromination positions (priority 3; reversed IUPAC priority). Bromine positions were solely listed by numbers without colon in between and without the prefixes *di-, tri-, tetra*- or *penta*- (e.g., 234-BP instead of 2,3,4-triBP). OH-TPs from HBBs were named bromophenols (BPs) and dihydroxylated isomers were named bromodiphenols (BDPs). In BDPs, the position of the second OH group was expressed as *ortho-*, *meta*-, or *para*- to the first hydroxyl residue, i.e., B*o*DP, B*m*DP, and B*p*DP (i.e., assigned to C2, C3, and C4, respectively). Hydroxylated metabolites of PBT and PBEB were labelled brominated methylphenols (BMePs) and brominated ethylphenols (BEtPs), respectively, with corresponding carbon positions of alkyl residues labelled *ortho-*, *meta*-, or *para* to C1 hydroxyl moiety (assigned to C2, C3, and C4, respectively). For instance, the 2,4,5-brominated congeners of the *meta*-isomers would be 245-B*m*DP, 245-B*m*MeP, and 245-B*m*EtP, respectively.

## Results and discussion

### Initial assessment and characterization of OH-TPs

UV irradiation with BTF as the solvent provided a much higher amount and variety of hydroxylated transformation products than toluene (details not shown). On the first glance, this was surprising because the hydrodebromination rate was previously shown to be much higher in toluene than in BTF (Klimm and Vetter 2021). Assumedly, in BTF which is a poorer H donor than toluene (Maul et al. 1999), the slower hydrodebromination rate most likely favored the formation of OH-TPs. This unpreceded observation was very helpful so that all evaluations were based on the use of BTF.

**Fig. 1:**
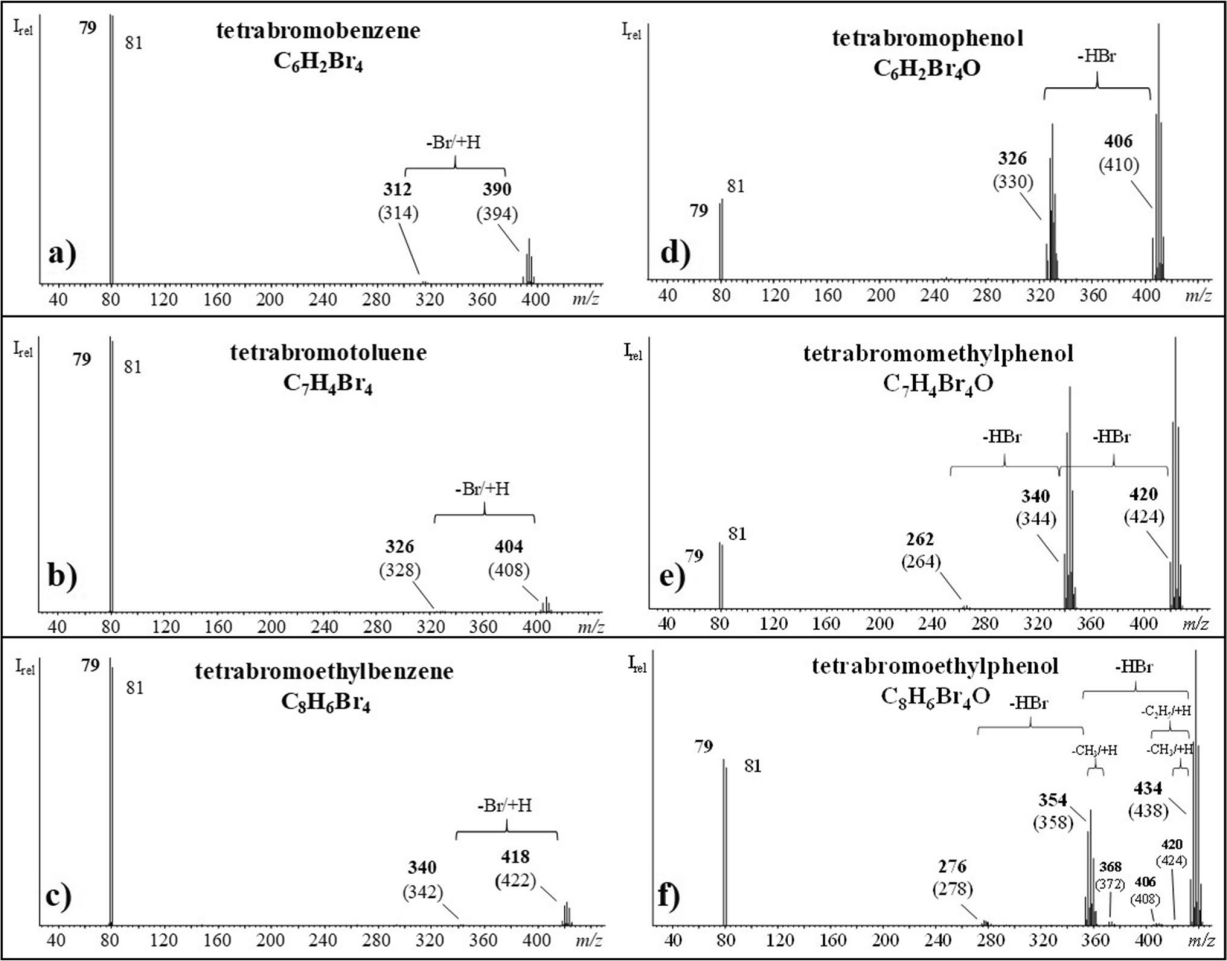
GC/ECNI-MS spectra of tetrabrominated (**a**–**c**) hydrodebrominated and (**d**–**f**) hydroxylated transformation products of hexabromobenzene (HBB), pentabromotoluene (PBT), and pentabromoethylbenzene (PBEB). Monoisotopic peaks are printed in bold while the corresponding base peaks are listed below them in parentheses

**Fig. 2:**
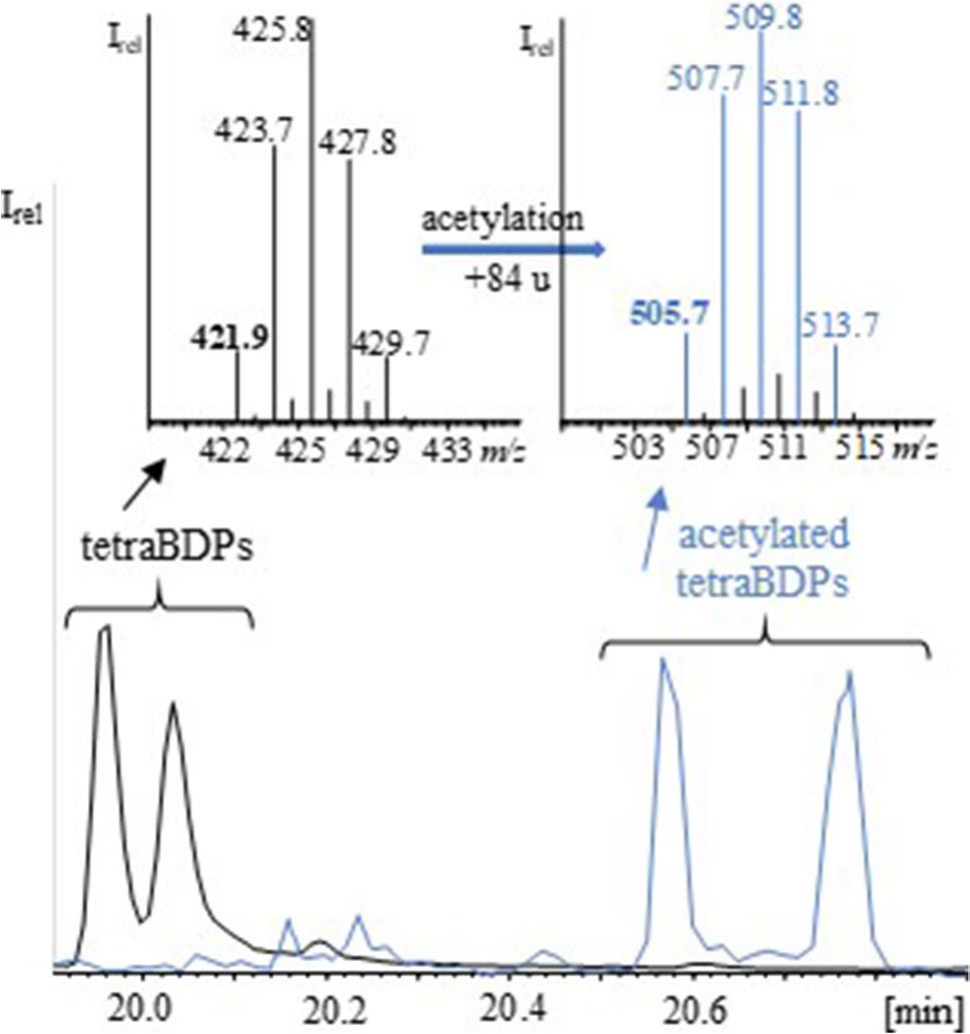
GC/ECNI chromatograms (bottom) and respective mass spectra of free tetrabromodiphenols (tetraBDPs) (black) and the corresponding diacetylated tetrabromodiphenols with M^−^ being shifted by 84 u to higher mass (blue)

GC/ECNI-MS analysis of silica gel fraction F3 of the UV irradiated sample solutions of all three cuBFRs (see the “Materials and methods” section) verified the presence of tri- and tetrabrominated OH-TPs. In addition, pentabromophenol (pentaBP) was detected in the case of HBB. GC/ECNI-MS spectra of the compounds in silica gel fraction 3 of the three cuBFRs showed the characteristic bromine isotope ions (*m/z* 79 and *m/z* 81) along with M^−^ (Fig. 1, right panels). The molecular ions were not only shifted by 16 u to higher mass but they also represented the base peak. This was in sheer contrast to the low abundant M^−^ of the corresponding hydrodebromination products with the same degree of bromination (Fig. 1). In addition, the GC/ECNI-MS spectra of the oxygenated TPs featured prominent [M-HBr]^−^ ([M-80]^−^) fragment ions (Fig. 1, right panels) while the corresponding hydrodebromination products showed low abundant [M-Br+H]^−^ ([M-78]^−^) fragment ions (Fig. 1, left panels). The presence of free (and thus derivatizable) hydroxyl groups in silica gel fraction F3 was clarified by acetylation of aliquots (Fig. S2). GC/ECNI-MS analysis of the resulting solutions confirmed shifts in retention times. Likewise, the acetylated M^−^ was higher by 42 u (which corresponds with the substitution of the phenolic hydrogen with a CH_3_CO moiety). In contrast to PBT or PBEB, UV irradiated solutions of HBB also featured dihydroxylated TPs (bromodiphenols, BDPs). Their authenticity could also be confirmed by acetylation. For instance, M^−^ of the acetylated tetrabrominated diphenol (tetraBDP) isomers was shifted by 84 u to higher mass compared to tetrabrominated benzenes (Fig. 2).

In order to omit additional expenditure by the derivatization step, subsequent evaluations were carried out with the free (non-acetylated) OH-TPs. In addition, the ratio of M^−^ to Br^−^ (M^−^/Br^−^) increased in the order bromobenzenes (7.8) < bromophenols (48) < bromodiphenols (83). Accordingly, all OH-TPs could be properly determined via M^−^, and the corresponding *m/z* values were implemented in the GC/ECNI-MS-SIM method (Tab. S1). Isomers could be assigned with the help of GC retention time data which was available for all OH-TPs including BDPs (Table 1) (Klimm and Vetter 2022). In agreement with the increasing molecular mass, cuBFRs and their OH-TPs eluted in the order Br_x_-TP << OH-Br_x_-TP << diOH-Br_x_-TP (BDPs, only in the case of HBB) < Br_x+1_-TP from the GC column (no overlap between the groups). UV irradiation of the cuBFRs was stopped and inspected after 10 min (1st UV irradiation, see the “Materials and methods” section). Then, OH-TPs were separated by silica gel fractionation and UV irradiated for a second time (10–50 min, 2nd UV irradiation, see the “Materials and methods” section). In this way, precursors could be separated which allowed to study the fate of the OH-TPs. However, and different to the precursor compounds (Klimm and Vetter 2021), reaction kinetics of the hydroxylated transformation products could not be determined in view of the varying response factors of individual compounds in GC/ECNI-MS chromatograms.

**Table 1:** Hydroxylated transformation products of the UV irradiation of HBB, PBT, and PBEBs in benzotrifluoride (BTF) along with GC/ECNI-MS data derived from Klimm and Vetter (2022)

Chemical name	Short Form	GC t_R_ (min)	m/z**
Brominated phenols (BPs) and diphenols (BDPs)			
2,4,6-tribromo-*m*-diphenol*	246-B*m*DP	17.42	344
2,4,5-tribromo-*m*-diphenol*	245-B*m*DP	17.69	344
4,5,6-tribromo-*m*-diphenol/2,3,5-tribromo-*p*-diphenol*	456-B*m*DP-/235-B*p*DP	17.73	344
2,3,4,5-tetrabromophenol	2345-BP	18.95	406
2,3,4,6-tetrabromophenol	2346-BP	19.06	406
2,3,5,6-tetrabromo-*p*-diphenol*	2356-B*p*DP	21.00	422
2,4,5,6-tetrabromo-*m*-diphenol*	2456-B*m*DP	21.11	422
2,3,4,5,6-pentabromophenol	23456-BP	22.36	484
Brominated methylphenols			
3,4,6-tribromo-*o*-methylphenol	346-B*o*MeP	16.54	342
3,4,5-tribromo-*o*-methylphenol	345-B*o*MeP	16.83	342
2,3,5-tribromo-*p*-methylphenol	235-B*p*MeP	16.89	342
2,4,5-tribromo-*m*-methylphenol	245-B*m*MeP	17.08	342
2,4,6-tribromo-*m*-methylphenol	246-B*m*MeP	17.18	342
2,3,6-tribromo-*p*-methylphenol	236-B*p*MeP	17.25	342
3,4,5,6-tetrabromo-*o*-methylphenol	3456-B*o*MeP	20.26	420
2,3,5,6-tetrabromo-*p*-methylphenol	2356-B*p*MeP	20.51	420
2,4,5,6-tetrabromo-*m*-methylphenol	2456-B*m*MeP	20.58	420
Brominated ethylphenols			
3,4,6-tribromo-*o*-ethylphenol	346-B*o*EtP	17.20	356
3,4,5-tribromo-*o*-ethylphenol	345-B*o*EtP	17.46	356
2,3,5-tribromo-*p*-ethylphenol	235-B*p*EtP	17.68	356
2,4,5-tribromo-*m*-ethylphenol	245-B*m*EtP	17.72	356
2,4,6-tribromo-*m*-ethylphenol	246-B*m*EtP	17.75	356
2,3,6-tribromo-*p*-ethylphenol	236-B*p*EtP	18.08	356
3,4,5,6-tetrabromo-*o*-ethylphenol	3456-B*o*EtP	20.76	434
2,3,5,6-tetrabromo-*p*-ethylphenol	2356-B*p*EtP	20.98	434
2,4,5,6-tetrabromo-*m*-ethylphenol	2456-B*m*EtP	21.05	434

*Compounds were not detected in parallel experiments with toluene (data not shown)

**For better readability, *m/z* values were rounded to nominal masses

### OH-TPs of HBB: bromophenols (BPs) and bromodiphenols (BDPs)

#### Bromophenols (BPs)

**Fig. 3:**
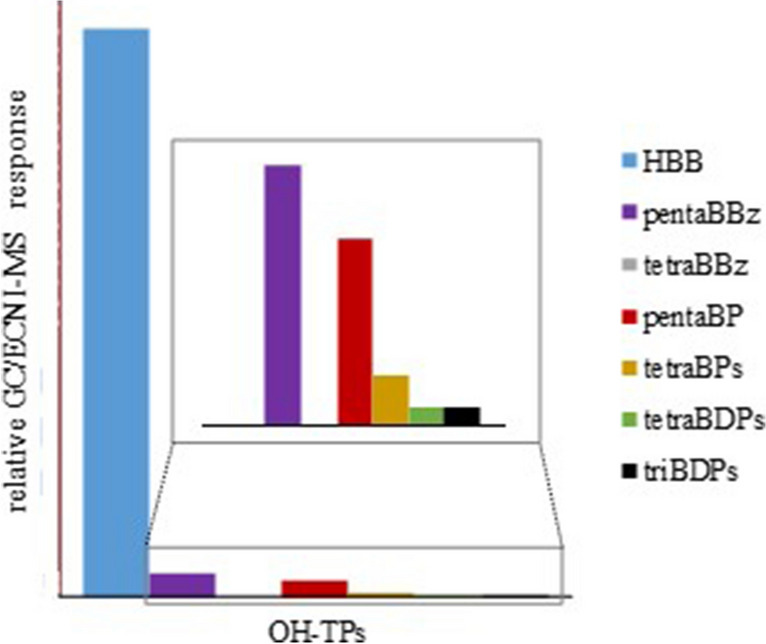
Relative and normalized GC/ECNI-MS-SIM responses (mean value) of formed transformation products (TPs), hydroxylated transformation products (OH-TPs), and dihydroxylated transformation products (diOH-TPs) and remaining share of hexabromobenzene (HBB) after the 1st UV irradiation (10 min) in benzotrifluoride (BTF). Evaluation was based on abundances of peak areas in the GC/ECNI-MS chromatograms and normalization to HBB which was set to 100%

After 10 min of UV irradiation of HBB, silica gel fraction F3 featured the predominant pentaBP along with two prominent tetraBPs, which were identified to be 2345-BP and 2346-BP (Fig. S3, Table 1). Due to the close GC elution of 2346-BP and 2356-BP (Δ*t*_R_ ~0.03 min) (Klimm and Vetter 2022), the presence (formation) of minute amounts of 2356-BP could not be fully excluded. Also, inspection of an acetylated sample aliquot did not indicate noticeable shares of 2356-BP, and its role was deemed negligible. At this point (1st UV irradiation, 10 min), the share of the transformation products (bromobenzenes, bromophenols, and bromodiphenols) was rather low and HBB was still predominant (Fig. 3). In order to get more insights into the fate of OH-TPs, a 2nd UV irradiation step was performed only with the OH-TPs present in silica gel fraction F3 (see the “Materials and methods” section). Hence, all bromobenzenes were separated and, subsequently, further products could only be formed from BPs and BDPs (which will be discussed below).

**Fig. 4:**
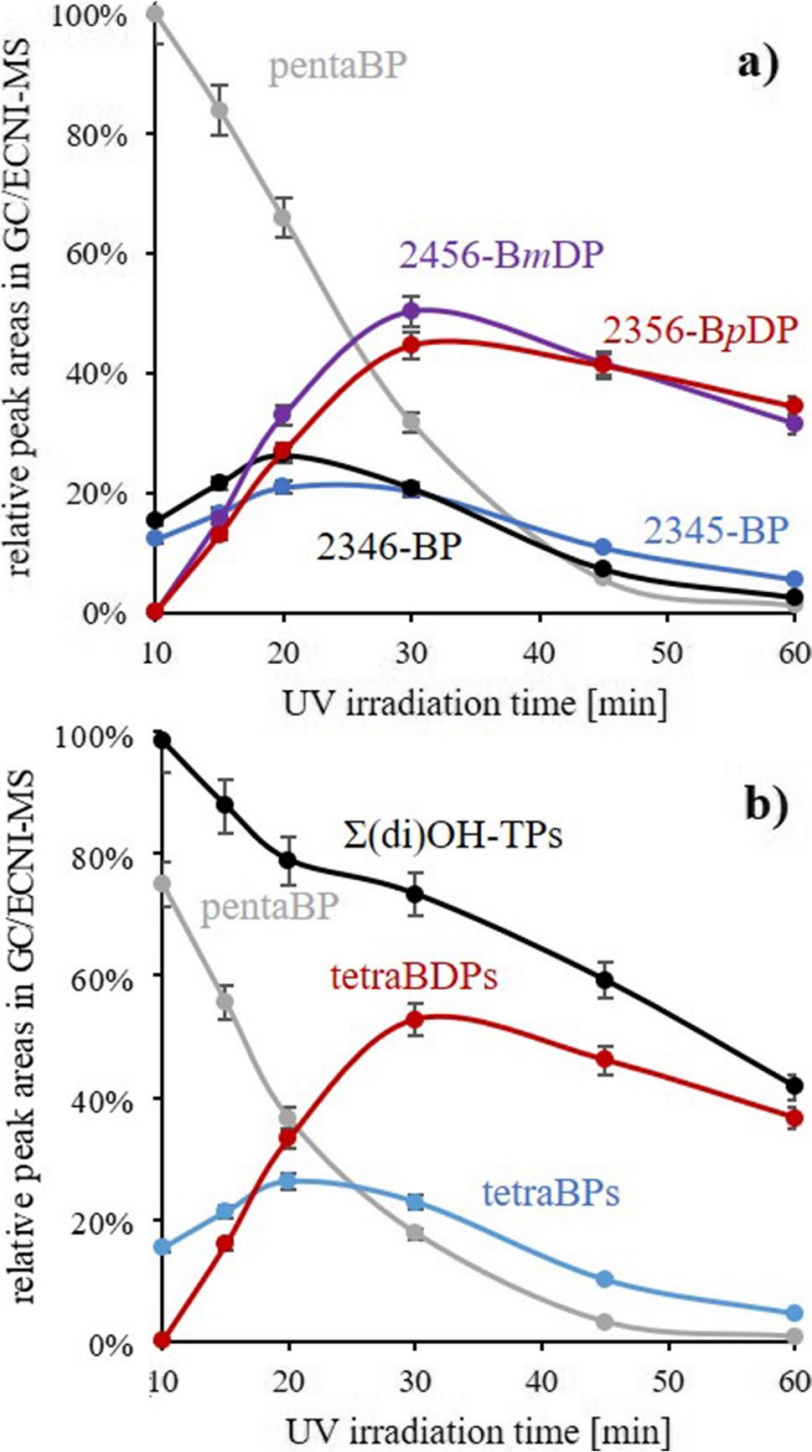
Formation and fate of hydroxylated transformation products (OH-TPs) of hexabromobenzene (HBB) initially UV irradiated in benzotrifluoride (BTF) for 10 min. Subsequently, the OH-TP fraction was isolated and UV irradiated a second time for a different period with **a** individual compounds and **b** grouped substance classes. Evaluation was based on the responses in the GC/ECNI-MS chromatograms

**Fig. 5:**
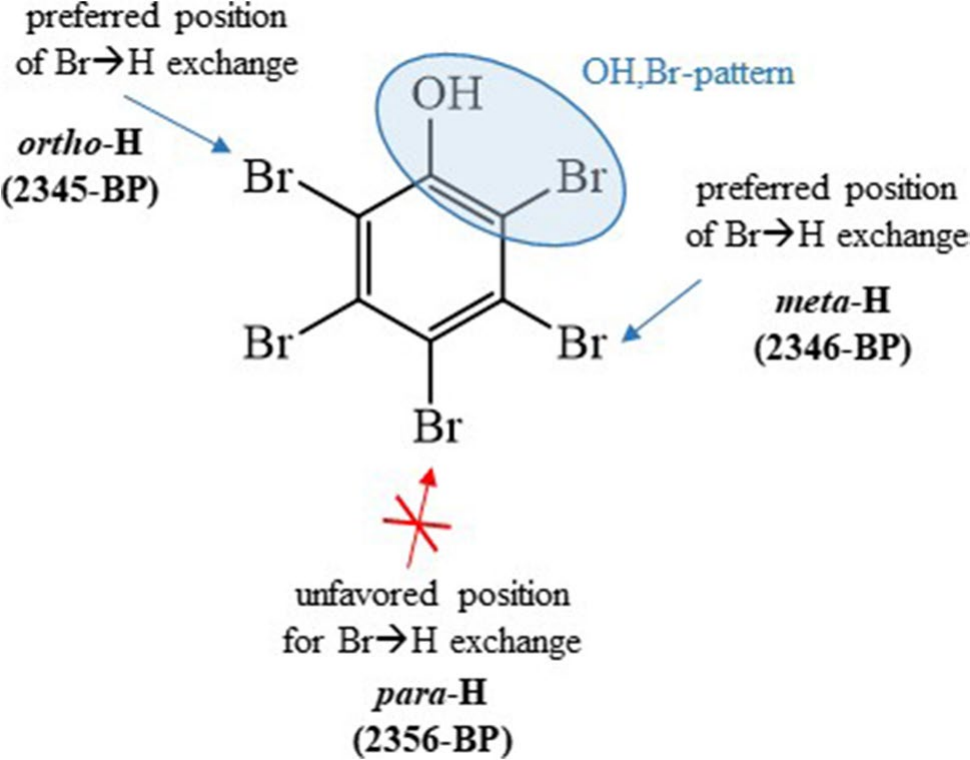
Hydrodebromination of pentabromophenol with positions indicating the formed (2345-BP and 2346-BP) and not formed (2356-BP) tetrabromophenols. Hydrodebromination was favored on positions with OH,Br pattern on α-position

Over the whole 2nd UV irradiation period (0–50 min; 10–60 min in total), 2345-BP (*ortho*-H) and 2346-BP (*meta*-H) evolved similarly (Fig. 4). However, before 30 min (when the precursor pentaBP was still relevant), 2346-BP was slightly more abundant, and from 30 min on, it was slightly less abundant than its isomer (Fig. 4a). This indicated that 2346-BP (*meta*-H) was more readily formed while 2345-BP (*ortho*-H) was more persistent. Apart from these details, Br➔H exchange in *ortho-* and *meta-*position was much more relevant than in *para*-position (little/no indications for 2356-BP). For BPs, Br➔H exchange was apparently favored vicinal to OH,Br-patterns, which leads to the formation of 2345-BP along with 2346-BP (Fig. 5). This concept is in agreement with the formation of nonabrominated PBDEs from BDE 209 whose relevance increased in the order BDE 206 (*ortho*-H) ≈ BDE 207 (*meta*-H) > BDE 208 (*para*-H) (Bendig et al. 2012).

Contrary to tetraBPs, triBPs were barely detected at any point of the 2nd UV irradiation. Only minute, noisy signals were visible in GC/ECNI-MS chromatograms at the corresponding retention times (data not shown). In accordance with observations of Bendig et al. (2014), injection of low amounts of BPs was accompanied with severe tailing so that it was impossible to assign any structure of the triBP isomers. The negligible role of triBPs was remarkable because both 2345-BP and 2346-BP still featured one Br substituent vicinal to a OH,Br-pattern. Apparently, the stability of the tetraBPs towards hydrodebromination was much higher than the one of pentaBP. Decreasing accessibility for UV degradation with decreasing degree of bromination is known from different substance classes (Wang et al. 2012; Ling et al. 2019). Bendig et al. (2013) suggested that a lower bromine density on the benzene ring is accompanied with a decreasing bathochromic shift and is thus a lower degree of hydrodebromination. At the end of the 2nd UV irradiation (60 min in total), pentaBP and the two tetraBP isomers were hardly detectable by GC/ECNI-MS (Fig. 4a).

#### Bromodiphenols (BDPs)

Low amounts of tetraBDPs and even triBDPs were already detected after the 1st UV irradiation of HBB (10 min) (Fig. 3). Especially the presence of triBDPs was surprising, since triBPs were hardly detectable at any point (see above). This also underlined the particular and promoting role of the OH group on UV transformation. However, at this point, triBDP amounts were lower than those of the tetraBPs (Fig. 3). During the 2nd UV irradiation step with the OH-TPs fraction, the abundance of the tetraBDPs permanently increased, and after ~20 min (10 min of the 2nd UV irradiation), they were the most abundant OH-TPs (Fig. 4b). Since bromobenzene supply was precluded during the 2nd UV irradiation, tetraBPs (1× Br➔OH exchange) and tetraBDPs (2× Br➔OH exchange) must be both transformation products of pentaBP which was steadily transformed (Fig. 4b). After 30 min, the amount of tetraBDPs reached its maximum, and at this point, they were twice as high abundant as the tetraBPs (Fig. 4b, cf. red vs. blue line). At this point, tetraBDPs already surmounted their precursor pentaBP, so that their formation was steadily depleting. As a consequence, tetraBDP amounts moderately decreased but stayed on a much higher level as those of tetraBPs which were only low abundant after 60 min UV irradiation (Fig. 4). This verified previous indications of the comparably high stability of tetraBDPs.

The tetraBDPs bear substituents on each carbon, and the three possible isomers differ only in the position of the second hydroxyl group which can be either in *ortho*-, *meta*-, or *para*-position relative to the first one. However, only 2356-B*p*DP and 2456-B*m*DP were detected (Table 1), while 3456-B*o*DP was not present at any point in the solutions. Similar to tetraBPs, one tetraBDP isomer was slightly more abundant in the first period (2456-B*m*DP) and the other one (2356-B*p*DP) in the second period (Fig. 4a). Accordingly, 2456-B*m*DP was suspected to be slightly more readily formed from pentaBP while 2356-B*p*DP appeared to be slightly more persistent than its isomer. Also in this case, Br➔OH exchange was slightly favored in *meta*-position. However, while Br➔H exchange (pentaBP➔tetraBP) was least likely in *para*-position (see above), Br➔OH exchange (pentaBP➔tetraBDP) was least likely in *ortho*-position which could be due to steric hindrance.

**Fig. 6:**
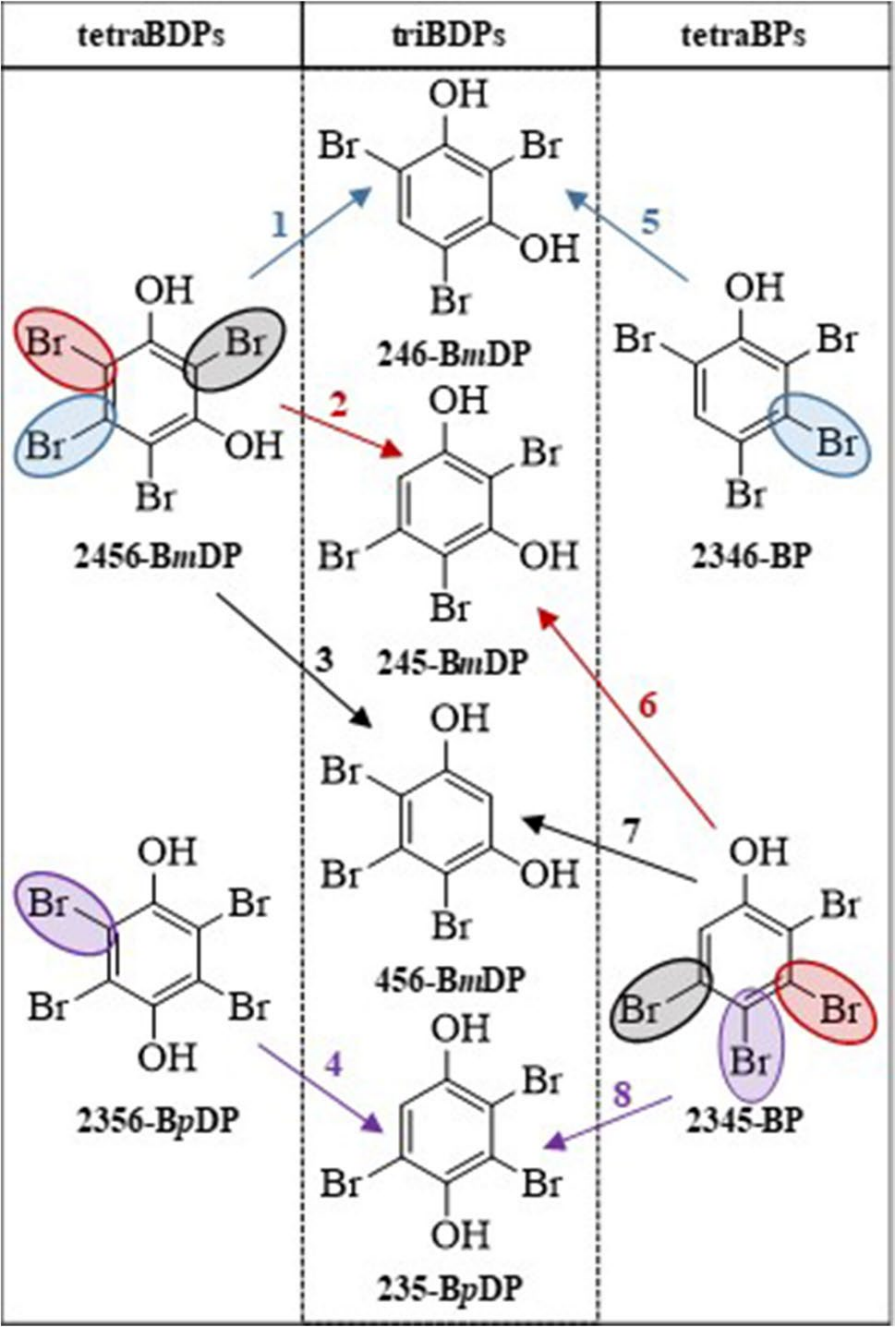
Observed tribromodiphenols (triBDPs) (central column) and the possible precursors, i.e., tetrabromodiphenols (tetraBDPs) via Br➔H exchange (left column) or tetrabromophenols (tetraBPs) via Br➔OH exchange (right column). Note that the latter two, 456-B*m*DP and 235-B*p*DP, were co-eluting in GC/ECNI-MS (see text)

Next to the two tetraBDPs, three peaks originating from triBDP isomers could be detected in the GC/ECNI-MS chromatograms. Two peaks could be unequivocally determined to be 246-B*m*DP and 245-B*m*DP. The third peak could be 456-B*m*DP and/or 235-B*p*DP (Table 1, very similar *t*_R_, resolution aggravated by tailing). These four isomers, i.e., three B*m*DP and one B*p*DP isomer, are those that can be formed from 2456-B*m*DP and 2356-B*p*DP (Fig. 6, left column). However, all four triBDPs can also be formed from 2345-BP and 2346-BP (Fig. 6, right column). As already discussed, tetraBPs were decreasing faster in abundance than tetraBDPs. Hence, it appeared more likely that the triBDPs were formed from 2345-BP and 2346-BP. Actually, triBDPs could be the main transformation products of tetraBPs. As already mentioned, both tetraBPs can be the precursors of the 246-B*m*DP, 245-B*m*DP (both verified) as well as 456-B*m*DP, and/or 235-B*p*DP (which co-eluted, see above) in the sample. However, formation of 456-B*m*DP was less likely because this could require an Br➔OH exchange vicinal to one unsubstituted carbon (reaction 7 in Fig. 6). Hence, the third peak in the GC/ECNI-MS chromatogram rather originated from 235-B*p*DP than from 456-B*m*DP.

The higher relevance of B*m*DP isomers agrees with the observation made above, namely the higher persistence of 2356-B*p*DP. In addition, absence of the two possible triB*o*DP isomers was not surprising since 2345-B*o*DP was not observed (see above) and formation from 2346-BP or 2345-BP would feature Br➔OH substitution vicinal to another OH group which features least favorability (Klimm and Vetter 2022). In this context, it is unfortunate that dibrominated compounds are hardly detectable by GC/ECNI-MS. Hence, further steps in the transformation route could not be explored. However, and as mentioned above, the susceptibility to UV transformation decreases with decreasing degree of bromination in accordance with the capability of biodegradation which also decreases with decreasing bromination level (Waaijers and Parsons 2016).

### OH-TPs of PBT: bromocresols or bromomethylphenols (BMePs)

#### tetraBMePs

**Fig. 7:**
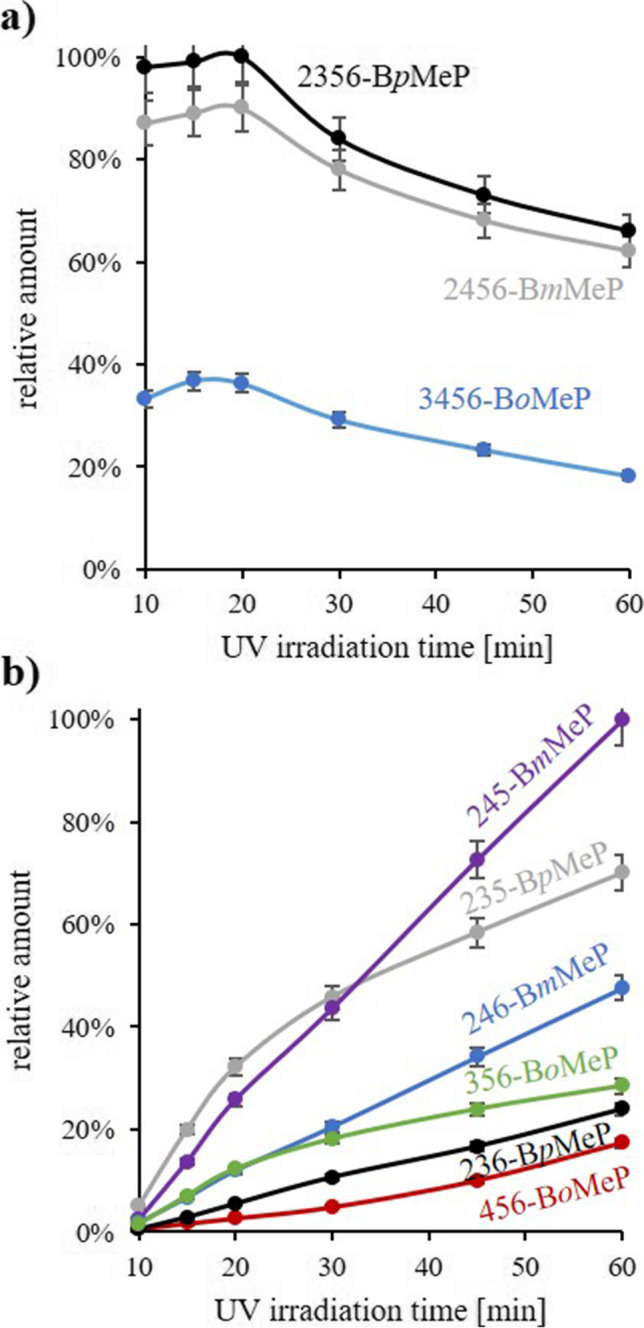
Formation and fate of the separated hydroxylated transformation products (OH-TPs) of pentabromotoluene (PBT) obtained during UV irradiation with **a** tetrabrominated methylphenols (tetraBMePs) and **b** tribrominated methylphenols (triBMePs). Compounds in **a** and **b** were individually normalized to 100% within the same bromination degree. Also, the starting point was set to 10 min UV irradiation time because this experiment was conducted with the OH-TPs obtained from the initial UV irradiation of PBT for 10 min. Evaluation was based on peak areas in the GC/ECNI-MS chromatograms

Notably, all three possible OH-tetraBTs were already detected after the 1st UV irradiation step of PBT. Yet, the amount of 3456-B*o*MeP was only about ~1/3 of the one of 2356-B*p*MeP and 2456-B*m*MeP (Fig. 7a, 10 min). Still, formation of 3456-B*o*MeP was unexpected because (the non-hydroxylated) 2,3,4,5-tetraBT (hydrodebrominated in *ortho*-position of the methyl group) was not formed in BTF (Klimm and Vetter 2021). On the one hand, this produced further evidence that the OH group was not introduced via H➔OH exchange (here: no formation of 3456-B*o*MeP from 2,3,4,5-tetraBT), but via direct Br➔OH exchange of PBT which is in accordance to observations made with HBB (see above). On the other hand, Br➔OH exchange vicinal to the methyl group could be facilitated due to less steric hindrance.

Continuation of the UV irradiation of silica gel fraction F3 seemed to lead to a slight increase of all three tetraBMeP isomers. Since no other precursors were present, this minute increase was most likely due to variations in the evaluation. However, after 10 min of the 2nd irradiation (20 min total), the amounts of all three tetraBMeP isomers were slightly decreasing. After normalization, their relevance decreased in the order 2356-B*p*MeP~3456-B*o*MeP > 2456-B*m*MeP (Fig. 7a). Still, after 1 h of UV irradiation, more than 60% of the three isomers could be detected. In contrast, hydrodebrominated photoproducts of PBT, i.e., tetraBTs, were already transformed after approximately 30–45 min (Klimm and Vetter 2021) which underlines the high persistence of the tetraBMePs.

#### triBMePs

Six triBMePs were already present in silica gel fraction F3 of PBT after the 1st UV irradiation (starting point *t*_0_ = 10 min), specifically two triB*m*MeP, triB*p*MeP, and triB*o*MeP isomers each (Table 1). The abundance of all six triBMePs increased throughout the 2nd UV irradiation (Fig. 7b). In terms of relevance, corresponding triBMePs slightly decreased in the order two triB*m*MeP isomers > two triB*p*MeP isomers > two triB*o*MeP isomers (each referring to the sum of the two triBMeP isomers). Klimm and Vetter (2022) postulated a favored hydrodebromination pathway for polybrominated aromatics which was observed in the present case, i.e., 245-B*m*MeP > 246-B*m*MeP for B*m*MePs, 235-B*p*MeP > 236-B*p*MeP for B*p*MePs and 356-B*o*MeP > 456-B*o*MeP for B*o*MePs, respectively (Fig. 7b).

### OH-TPs of PBEB: bromoethylphenols (BEtPs)

#### tetraBEtPs and triBEtPs

UV irradiation of PBEB resulted in polybrominated ethylphenols (BEtPs). Unlike PBT, GC/ECNI-MS fragment ions were also formed in the ethyl moiety in form of [M-15]^−^ and [M-29]^−^ (Fig. 1). Altogether, the isomer pattern corresponded well with the one of PBT (Tab. 1). However, triBEtPs peaked between 20 and 30 min followed by a decrease to 20–50% of this maximum after 60 min of UV irradiation (data not shown). Apparently, the longer alkyl residue had an influence on the transformation rate.

## Conclusions

UV irradiation of HBB, PBT, and PBEB generated polar transformation products in each case. In this context, the use of BTF turned out to be crucial for the key-findings in this study. Compared to toluene, the low hydrodebromination rate of the present analytes in BTF (Klimm and Vetter 2021) was most likely the key for the remarkably higher formation rate of OH-TPs. Therefore, the use of BTF is highly recommended to be tried when polar transformation products are going to be studied in laboratory experiments. This solvent selection was also important for the discovery that the UV transformation HBB even resulted in dihydroxylated metabolites that were found to be more stable in the laboratory experiment than the corresponding onefold hydroxylated TPs. However, little is known about their environmental behavior. Yet the noticed (comparably) high stability indicates that more research should be directed towards these transformation products. In this context, it is important to note that standard analysis protocols frequently do not cover these more polar compounds. However, first generation BFRs like PBDEs (and meanwhile banned) are known to form OH-TPs during UV irradiation (Lacorte et al. 2010). For these related compounds, it has already been shown that they can be found ubiquitous in the environment (Mas et al. 2007) and that they bear an increased ecological risk compared to their precursors due to their altered physiochemical properties (Zhao et al. 2015; Rayne and Forest 2010).

The higher polarity of formed OH-TPs of HBB, PBT, and PBEB indicated that they may not be enriched in the human body. In the environment, in turn, the introduced hydroxyl group(s) will increase the aqueous solubility and decrease the volatility. This will cause a shift from air transport to transport in the water phase. On the long term, these contaminants could also enter the drinking water reservoirs. Therefore, their removal in waste water treatment plants would be desirable and should be explored. Moreover, in contact with microorganisms, they could be also transferred into the corresponding anisoles and guaiacols. Especially bromoanisoles could show a higher potential for bioaccumulation. Therefore, the formation and persistence of the compounds as well as several research gaps need to be filled in the future as they could be important for the assessment of the safety of these and related BFRs.

### Supplementary information

ESM 1 The online version contains supplementary data available on the Springer website. (DOCX 378 kb)

## Data Availability

All relevant data is included in the manuscript or listed in the supplementary material.
