# Supplementary material for: Hydroxylated transformation products obtained after UV irradiation of the current-use brominated flame retardants hexabromobenzene, pentabromotoluene, and pentabromoethylbenzene
Source: Environ Sci Pollut Res Int. 2023 Nov 2;30(56):118556–66. doi: 10.1007/s11356-023-30566-w (PMC10697972; doi:10.1007/s11356-023-30566-w)
Supplement: Supplementary file 1 — The online version contains supplementary data available on the Springer website. (DOCX 378 kb) [file 11356_2023_30566_MOESM1_ESM.docx]

**Supplementary information for:**

**Hydroxylated transformation products obtained after UV irradiation of the current-use brominated flame retardants hexabromobenzene, pentabromotoluene and pentabromoethylbenzene**

*Environmental Science and Pollution Research*

Alexandra Klimm, Walter Vetter^*^

University of Hohenheim, Institute of Food Chemistry (170b), Garbenstraße 28, D-70599 Stuttgart, Germany

^*^ Corresponding author

Walter Vetter

Phone: +49 711 459 24016

Fax: +49 711 459 24377

Email: walter.vetter@uni-hohenheim.de

Content (for details see next page): 1 table and 3 figures

**Content**

| page S3 | Tab. S1: Time windows and ions measured by GC/ECNI-MS-SIM |
| --- | --- |
| page S4 | Fig. S1: Chemical structures of HBB, PBT and PBEB |
| page S5 | Fig. S2: GC/ECNI-MS chromatograms and MS of OH-TPs and their acetates |
| page S6 | Fig. S3: GC/ECNI-MS chromatograms of UV irradiated HBB, pentaBBz and pentaBP |
|  |  |
|  |  |

**Tab. S1:** GC/ECNI-MS-SIM time windows and respective measured ions according to Klimm and Vetter.^[[1]](#footnote-1)^ SIM 1 was used for hydroxylated transformation products of hexabromobenzene (HBB), SIM 2 for pentabromotoluene (PBT) and SIM 3 for pentabromoethyl benzene (PBEB) with BC-2 as internal standard (ISTD).

| **Time window [min]** | **SIM 1 ions [*m/z*]** | **Target compounds** |
| --- | --- | --- |
| 9-18 | 79/81, 328-334, 344-350 | triBPs/triBDPs |
| 18-22 | 79/81, 406-412, 422-428 | tetraBPs/tetraBDPs |
| 22-24 | 79/81, 484-492 | pentaBP |
| 24-40 | 79/81, 512-520 | BC-2 (ISTD) |
| **Time window [min]** | **SIM 2 ions [*m/z*]** | **Target compounds** |
| 15-19 | 79/81, 342-348, 358-366 | triBMePs/triBMeDPs |
| 19-24 | 79/81, 420-428 | tetraMeBPs |
| 24-40 | 79/81, 512-520 | BC-2 (ISTD) |
| **Time window [min]** | **SIM 5 ions [*m/z*]** | **Target compounds** |
| 16.5-20 | 79/81, 356-362, 372-380 | triBEtPs/triBEtDPs |
| 20-24 | 79/81, 434-442 | tetraEtBPs |
| 24-40 | 79/81, 512-520 | BC-2 (ISTD) |

**Fig. S1:** Chemical structures of (a) hexabromobenzene (HBB), (b) pentabromotoluene (PBT) and (c) pentabromoethylbenzene (PBEB).

**Fig. S2**: GC/ECNI chromatograms and respective mass spectra of free hydroxylated TPs (black), namely OH-pentaBBz (a), OH-tetraBTs (b) and OH-tetraBEB (c) and the corresponding acetates with M^-^ shifted by 42 u to higher mass (blue).

**Fig. S3:** GC/ECNI-MS chromatograms of the UV irradiation of (a) hexabromobenzene (HBB), (b) pentabromobenzene (pentaBBz) and (c) pentabromophenol (pentaBP) in benzotrifluoride (BTF).

1. Klimm, A.; Vetter, W. Synthesis and evaluation of hydroxy‑ and dihydroxy brominated benzenes, methyl- and ethylbenzenes: Potential metabolites of current-use brominated flame retardants. *J. Chromatogr. A* **2022,** *1673*, 463109 [↑](#footnote-ref-1)
